# Aberrant Right Subclavian Artery Incidentally Detected in Eight Adult Dogs: CT Findings and Possible Association with Intermittent Regurgitation

**DOI:** 10.3390/life16101694

**Published:** 2026-10-09

**Authors:** Niculae Tudor, Vlad Traian Lupu, Nicoleta Andreea Mincă, Andrei Mario Codreanu, Poliana Tudor, Cosmin Mihai Rada, Cristina Fernoagă

**Affiliations:** 1 Clinical Sciences II Department, Faculty of Veterinary Medicine, University of Agronomic Sciences and Veterinary Medicine of Bucharest, 011464 Bucharest, Romania; niculae.tudor@fmvb.usamv.ro (N.T.); cosmin.rada@fmvb.usamv.ro (C.M.R.); 2 Link-Vet Veterinary Clinic, 021624 Bucharest, Romania; vlad.lupu@hotmail.com; 3 Clinical Sciences I Department, Faculty of Veterinary Medicine, University of Agronomic Sciences and Veterinary Medicine of Bucharest, 011464 Bucharest, Romania; andrei-mario.codreanu@fmvb.usamv.ro (A.M.C.); cristina.fernoaga@fmvb.usamv.ro (C.F.)

**Keywords:** aberrant right subclavian artery, dogs, computed tomography, oesophageal compression, regurgitation, vascular anomaly

## Abstract

An aberrant right subclavian artery (ARSA) is an uncommon vascular anomaly that may cause oesophageal compression and clinical signs such as regurgitation. This exploratory case series describes the computed tomography (CT) findings of isolated ARSA in eight adult dogs (aged 3–13 years) examined for unrelated conditions. Contrast-enhanced CT images were evaluated for the origin and course of the aberrant artery, its relationship with the oesophagus and trachea, and evidence of oesophageal compression or secondary changes. In all dogs, the right subclavian artery originated directly from the aortic arch and followed a course from left to right, passing dorsal to the oesophagus and trachea, producing mild focal oesophageal compression. No oesophageal dilation or abnormal intraluminal accumulation of gas or fluid was identified. Following CT identification of ARSA, targeted questioning of the owners revealed a history of rare or intermittent regurgitation in six of the eight dogs (75%). Although a causal relationship between ARSA and regurgitation could not be established, these findings suggest that isolated ARSA may be associated with subtle oesophageal compression in adult dogs. The incidental identification of this vascular anomaly on CT may be relevant when evaluating dogs with intermittent regurgitation.

## 1. Introduction

Vascular ring abnormalities (VRAs) are congenital malformations of great vessels that can result from embryological development disturbances of aortic arch regression [1,2,3]. Depending on the specific vascular configuration, these abnormalities can surround or compress the trachea and oesophagus to varying degrees, thus causing digestive or respiratory dysfunction. Several types of VRA have been described in dogs, which may or may not be associated with clinical manifestations [1,3,4,5]. Among imaging examinations, CT examination, especially CT angiography, provides a detailed characterisation of the origin and course of abnormal vessels, as well as their anatomical relationship with the oesophagus and trachea, allowing differentiation between the various vascular configurations [1,2,4,6,7,8].

Normally, in dogs, the right subclavian artery arises from the brachiocephalic trunk [1]. However, sometimes the aberrant right subclavian artery (ARSA), also referred to in human medicine as “arteria lusoria” [9], can occur, which is a congenital vascular abnormality, characterised by abnormal origin and trajectory. In dogs, ARSA originates directly from the aortic arch or descending aorta, and then follows a dorsal trajectory (usually in a retroesophageal position), before continuing to the right thoracic limb [1,6,10]. This abnormal anatomical distribution can cause focal oesophageal compression, although its degree and clinical consequences appear to vary [2]. VRA is considered a “vascular ring” abnormality, which is less common in dogs [11] and those clinically affected may experience regurgitation or other signs associated with oesophageal dysfunction [2,6,12,13]. However, ARSA can also be detected incidentally in dogs that do not show clinical signs of gastrointestinal or respiratory impairment [1,2].

According to the literature, the reported prevalence of ARSA ranges from 0.8% [7] to 1.6% [1], while its potential clinical significance in dogs has only recently begun to be systematically investigated [2]. It has been found that, in most situations, it represents an incidental finding and remains clinically silent [2]. However, under certain circumstances, this vascular abnormality may become clinically relevant [2]. Its abnormal trajectory, over the oesophagus and trachea, can sometimes produce oesophageal compression manifested predominantly by oesophageal transit disorders and regurgitation [6,13]. These results indicate that the anatomical presence of ARSA does not necessarily imply clinically significant compression of the oesophagus and that the relationship between vascular abnormality, oesophageal changes and clinical signs remains incompletely defined. Furthermore, sometimes, oesophageal compression is not only caused by the presence of ARSA, but can result from the combination of several vascular structures or a complex vascular ring [3,13].

Although esophageal transit disorder associated with ARSA has been reported and manifests clinically, including cases presenting with regurgitation [6,13], less detail is known about adult dogs in which ARSA is incidentally detected and causes only mild oesophageal compression with no secondary esophageal changes such as its dilation. In particular, it remains unclear whether such discrete anatomical changes may be associated with intermittent gastrointestinal signs that are not evident or considered relevant at the time of initial clinical examination. Therefore, the aim of this exploratory case series was to characterise the CT features of incidentally detected ARSA in eight adult dogs and to assess the reported occurrence of intermittent regurgitation in the absence of CT evidence of significant oesophageal compression.

## 2. Materials and Methods

The medical records of canine patients registered in the database of the Link-Vet Bucharest clinic, between 2020 and 2025, were reviewed in order to identify possible cases of ARSA. The criteria for inclusion of canine patients in the study were as follows: the existence of a medical file with a complete clinical history; the existence of a complete CT examination (performed before and after the administration of the contrast agent) of the cervico-thoraco-abdominal region, performed based on the referral note issued by the attending physician; the presence of the aberrant right subclavian artery diagnosed based on the CT scan.

The identification data of the patients, including breed, age, sex and body weight, were taken from the medical records.

The CT scan was performed according to a previously published protocol [14]. The canine patients were subjected to a diet (fasting of at least 10 h) and a liquid-only diet (water-only fasting) for at least 2 h before CT examination, which was performed using a CT scanner—GE HealthCare Revolution CT, Waukesha, WI, USA equipment. The canine patients were placed in sternal recumbency, using radiolucent devices for positioning. The anaesthetic protocol applied was identical for all dogs [8,14,15]. Premedication was performed with butorphanol at a dose of 0.2–0.3 mg/kg IV, midazolam 0.2–0.3 mg/kg IV, medetomidine 4–8 mcg/kg IV, followed by induction with propofol 1–6 mg/kg, intubation, maintenance with isoflurane and 100% oxygen, monitoring throughout the procedure [14,15]. Native transverse, sagittal, and dorsal images of the anatomical regions recommended by the attending physicians who requested CT scan were acquired. Subsequently, post-contrast images were acquired, following the administration of Iopamidol (Iopamiro 370 mg/mL, Bracco Imaging SPA, Milan, Italy) at a dose of 2 mL/kg through the cephalic vein using an automated power injector (Nemoto Smart Shot Alpha-Japan, Tokyo, Japan).

Following the incidental identification of ARSA on CT, the owners were specifically asked during the completion of the clinical history whether they recalled any previous episodes of regurgitation during the dog’s lifetime. The information obtained was documented in the medical file of each patient.

Due to the exploratory nature of the study, approval from an institutional ethics committee was not required. The study was conducted using clinical data obtained during routine veterinary care and documented in the animals’ medical records. No experimental procedures or interventions were performed specifically for the purposes of the study. Therefore, approval from an ethics committee was not required.

## 3. Results

Eight canine patients with incidentally detected ARSA were included in the present exploratory case series. The dogs were three to 13 years old (mean, 8.0 years; median, 8.5 years) and weighed six to 31 kg. Five canine patients were females and three were males. The study group included dogs of different breeds, including two Border Collies, two French Bulldogs, one German Shepherd Half-breed, one Bichon, one Boxer, and one American Staffordshire Terrier. Detailed individual clinical characteristics and indications for CT examination are presented in Table 1.

During the study period, 2569 clinical CT examinations were performed. All examinations were reviewed, and eight cases with an aberrant right subclavian artery (ARSA) were identified and subsequently included in the present exploratory clinical case series based on the predefined inclusion criteria. No additional cases with suspected or confirmed ARSA were excluded because of incomplete medical records or failure to meet the predefined inclusion criteria.

Animals were referred for CT examination due to clinical conditions unrelated to a suspicion of vascular or oesophageal disease. Indications included neurological, musculoskeletal or respiratory disorders. The main CT findings corresponded to the clinical indications for examination and ARSA was identified as an incidental finding in all eight canine patients. In four dogs (Cases 2, 3, 4, 7), CT examination was performed mainly due to the presence of vertebral changes or neurological disorders, including intervertebral disc disease, deforming spondylosis, congenital vertebral malformations and neurological deficits. In the other canine patients, examination was performed due to clinical signs of respiratory, pulmonary (Cases 5, 6) or upper respiratory tract (Cases 1, 8).

**Figure 1:**
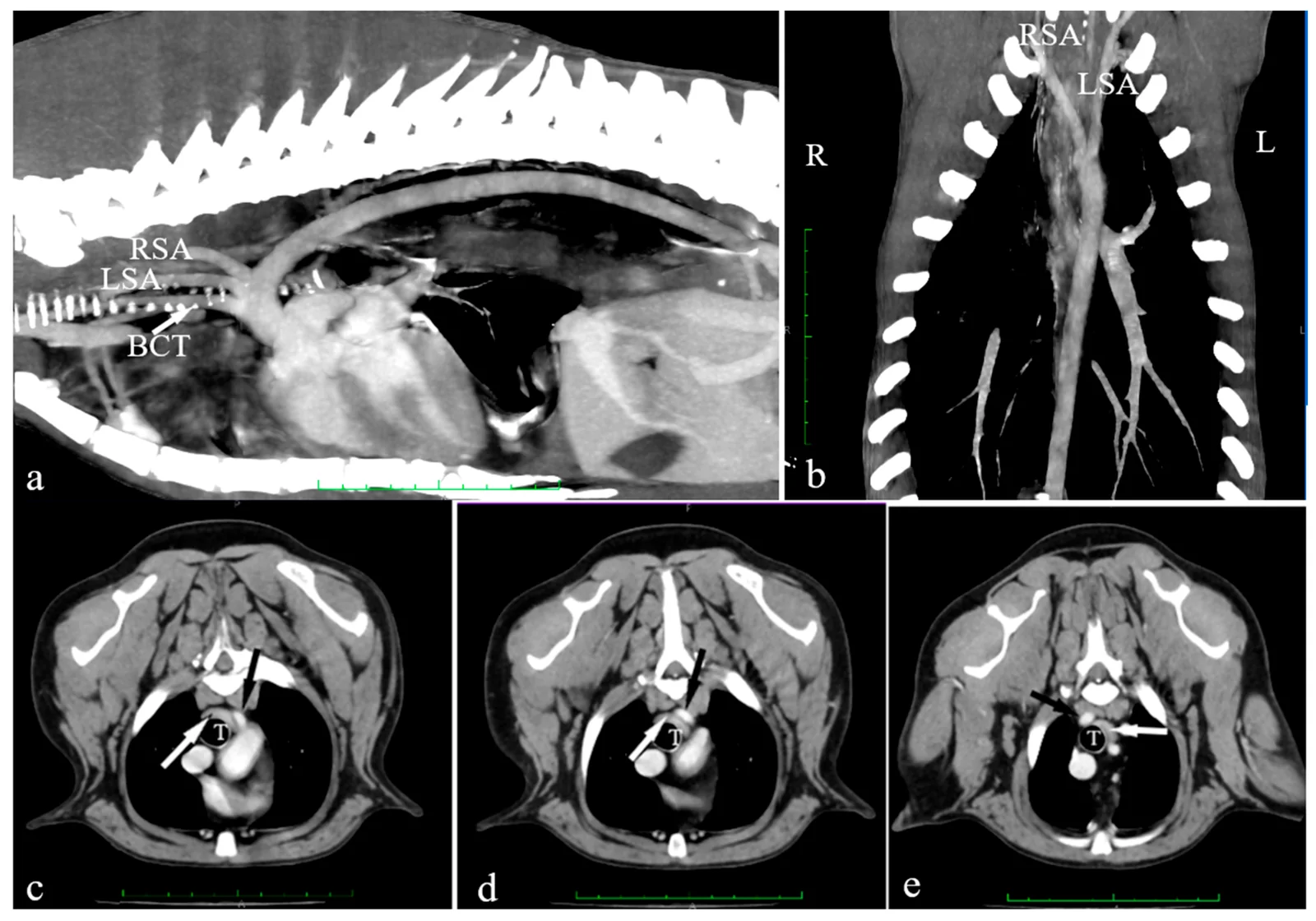
Post-contrast CT images reconstructed in a soft-tissue window in the sagittal (**a**), dorsal (**b**), and transverse (**c**–**e**) planes. (**a**,**b**) Case 7: sagittal and dorsal multiplanar reconstructions demonstrating the aberrant origin of the right subclavian artery (RSA) directly from the aortic arch, in relation to the brachiocephalic trunk (BCT) and left subclavian artery (LSA). (**c**–**e**) Case 8: consecutive transverse images illustrating the course of the aberrant right subclavian artery (black arrows), arising from the left aspect of the aortic arch (**c**), passing dorsally to the oesophagus (white arrows) and trachea (T) (**d**), and continuing towards the right side (**e**), resulting in mild focal oesophageal compression.

In all cases included in the present exploratory case series, the right subclavian artery was identified as originating directly from the aortic arch (Figure 1). The artery followed a trajectory adjacent to the left lateral aspect of the trachea and oesophagus, subsequently passing the dorsal obliquely over these structures before continuing its normal cranial route, on the right side, to the right thoracic limb. In all eight dogs, a slight imprint/compression of the oesophagus was observed, associated with the aberrant vascular pathway. Despite the presence of this vascular imprint, no cranial or caudal oesophageal dilation was identified from the point where the aberrant artery crosses the oesophagus. Also, the oesophageal lumen did not show detectable accumulations of gas or fluid in any of the eight dogs at the time of CT examination. No other vascular abnormalities in the aortic arch were identified in any of the dogs. The individual CT characteristics of ARSA and the associated oesophageal findings are summarised in Table 2.

At the time of CT examination, none of the canine patients had been referred for consultation due to the suspicion of oesophageal disease or persistent gastrointestinal clinical signs. However, targeted owner questionnaires revealed a history of postprandial regurgitation in six of the eight patients (75%), although these episodes had not been adequately investigated at the time. Although the episodes were first reported at 6–8 months of age, they were mild and transient, and their frequency gradually decreased over time. At that time, regurgitation episodes were attributed to rapid food ingestion, gastroesophageal reflux, conformation in brachycephalic patients, or inadequate food ingestion during walks. Accordingly, in most cases, symptomatic treatment was provided, resulting in the resolution of the clinical signs, without further paraclinical investigations being performed. No significant changes in the segments of the digestive tract were identified during previous clinical examinations in dogs for which such information existed. In one of the situations (Case 2), the owner had adopted the animal approximately two years prior to this research and could not provide reliable information on the gastrointestinal history, and in Case 5, it was found that the patient did not have a reported history of clinically significant gastrointestinal disease.

## 4. Discussion

This exploratory case series describes the CT features of an aberrant right subclavian artery (ARSA), incidentally identified in eight adult canine patients, and assesses its possible clinical relevance in relation to the gastrointestinal signs reported in their medical history. The most consistent finding was the remarkably similar anatomical appearance of the vascular abnormality in all dogs. In each case, ARSA originated directly from the aortic arch, followed a retroesophageal pathway, and caused mild focal compression of the oesophagus. However, none of the patients experienced cranial oesophageal dilation from the site of compression, and no accumulation of gas or fluid was identified in the esophageal lumen. In addition, no other vascular abnormalities of the aortic arch were detected. Despite the absence of CT evidence of significant oesophageal obstruction, six of the eight canine patients had a history of rare or intermittent regurgitation, as reported by their owners during the clinical evaluation. The coexistence of these findings constitutes the main aspect of interest of this series and raises the question of whether apparently mild ARSA-associated oesophageal compression could have discrete or intermittent functional consequences in some dogs.

Aberrant right subclavian artery is a congenital anomaly of the aortic arch in which the right subclavian artery originates separately from the aortic arch and passes dorsally through the oesophagus and trachea, before continuing its trajectory to the right thoracic limb [1]. This anatomical configuration may result in partial circumferential and variable compression of the oesophagus and is therefore classified as a VRA in dogs. However, the presence of ARSA does not necessarily imply clinically significant oesophageal obstruction, as previously demonstrated [1,2].

This interpretation was recently reinforced by a large study that specifically evaluated ARSA in dogs [2], identifying this vascular abnormality in 1.2% of dogs undergoing a CT examination with contrast agent. In addition, within the large group of 37 dogs with ARSA in that study, the vascular abnormality was most likely considered incidental in 75.6%, potentially clinically relevant in 21.6% and clinically relevant in 2.7% of them. It is important to note that when ARSA was considered clinically relevant, oesophageal compression was present at the site where the aberrant vessel crossed the oesophagus, accompanied by a cranial oesophageal dilation containing gas and fluid. Oesophageal dilation associated with compression has also been identified more frequently among dogs in which the vascular abnormality was considered potentially clinically relevant [2,5]. These observations suggest that the mere anatomical presence of ARSA is insufficient to establish clinically significant correlations and that secondary oesophageal changes are important in the interpretation of this vascular abnormality.

The present cases differ from the clinically manifested ARSA, previously described in [5,13], in one important aspect. Although mild focal oesophageal compression was visible in all eight dogs, none showed oesophageal dilation or gas/fluid accumulation. These findings provide no evidence (by CT) of persistent or significant mechanical compression at the time of examination. This distinction is clinically relevant, as oesophageal dilation proximal to the site of vascular constriction is a frequent imaging finding in vascular ring anomalies with functional clinical significance [16].

Therefore, the absence of secondary oesophageal dilation in the dogs in this study argues against the existence of a severe or clinically significant obstruction. However, this does not necessarily demonstrate that the anatomical compression was completely devoid of functional consequences. This distinction is particularly relevant in the study group, as six of the eight dogs (75%) had a history of rare or intermittent regurgitation reported by their owners during the clinical evaluation. The reported episodes were generally rare, intermittent or transient, and not persistent, and none of the dogs had been referred for consultation to investigate a suspected oesophageal disease. Consequently, the symptomatology recorded in these animals differs significantly from the typical clinical picture of clinically relevant vascular ring abnormalities, in which persistent postprandial regurgitation, poor development or poor body maintenance often led to diagnostic investigations from an early age [13]. However, previous studies have reported the presence of adult-onset regurgitation in patients presenting with oesophageal compression associated with vascular abnormalities, including ARSA [6,13,17,18]. These reports indicate that congenital vascular abnormalities may occasionally become clinically apparent later in life, rather than exclusively around the time of weaning. However, the dogs in the present study should not be considered equivalent to previously reported clinically affected cases. In our series, ARSA was not identified during previous investigations, but incidentally, much later, during CT examinations performed for unrelated conditions. Furthermore, the degree of oesophageal compression was mild and no secondary oesophageal dilation was observed. Therefore, the reported occurrence of regurgitation cannot be considered clear evidence that ARSA was responsible for these clinical episodes.

Various alternative explanations for the occurrence of regurgitation have been identified in some dogs in the present study. Some episodes had previously been associated with rapid food ingestion, brachycephalic conformation, a suspected gastroesophageal reflux or non-specific gastrointestinal disorders. In addition, as the clinical history was based on owner-reported information, the exact nature of some episodes could not always be independently verified, and the distinction between regurgitation and vomiting may have been imprecise in the descriptions provided by the owners. For these reasons, the finding that six out of eight dogs had a history of regurgitation should be interpreted as an association rather than as evidence of causation.

However, the occurrence of owner-reported regurgitation in six of the eight dogs included in the present study is noteworthy and raises the possibility that even mild oesophageal compression associated with ARSA may have intermittent functional relevance. A relevant comparison is provided by the recent study by Jones et al. [2], in which most cases of ARSA were considered incidental, but a smaller subgroup could not be classified with certainty as lacking clinical relevance. The authors specifically concluded that since clinically relevant cases were rare, no single imaging criteria could determine with certainty the clinical significance of ARSA. The current findings highlight the discrepancy between the anatomical and clinical features observed in these patients. Although all eight dogs showed anatomical compression of the oesophagus, none had evidence of oesophageal dilation, while six had a history of intermittent regurgitation.

One possible explanation is that the morphological severity observed during a static CT examination may not fully reflect oesophageal function. Although CT accurately depicts the anatomical relationship between the abnormal vessel, oesophagus, and trachea, it only provides a momentary image of these structures at the time of examination. Swallowing and oesophageal transit are dynamic processes. Therefore, it is plausible that a mild fixed narrowing would have minimal or no consequences in most situations, but would disrupt the passage of the food bolus under specific conditions, such as rapid ingestion or passage of a larger or more solid food bowl. This remains a hypothesis, as dynamic oesophageal function was not evaluated in the dogs in this study. The available data therefore do not demonstrate the existence of an intermittent obstruction and should not be interpreted as evidence that a slight compression necessarily causes regurgitation.

Consequently, in dogs, the clinical significance of ARSA appears to lie on a spectrum ranging from incidental findings with no clinical evidence of oesophageal compression [1] to clinically significant cases associated with oesophageal compression and proximal dilation containing gas or liquid [2]. Therefore, the dogs in this study, characterised by mild oesophageal compression without secondary dilation, could represent an intermediate morphological phenotype, the clinical significance of which remains uncertain.

The absence of additional vascular abnormalities of the aortic arch in all eight dogs represents another relevant finding. Vascular ring abnormalities constitute a heterogeneous group of congenital vascular configurations [1,3,18], and their effects on the oesophagus depend on the complete vascular anatomy rather than the presence of a single abnormal vessel. Therefore, CT angiography is valuable not only for the identification of ARSA, but also for determining whether other abnormal vessels or configurations of the aortic arch contribute to oesophageal or tracheal compression [1,3]. The dogs in the present study had an isolated ARSA, with no other vascular abnormalities detected in the aortic arch. This observation reinforces the anatomical association between the aberrant vessel and the mild focal oesophageal imprint observed on CT examination, but does not demonstrate that this degree of compression was sufficient to cause clinical manifestations. However, it emphasises the importance of differentiating an isolated ARSA, which causes limited oesophageal deformity from more complex vascular configurations, capable of causing significant mechanical obstruction.

Also noteworthy is the age of the canine patients in the present series. All were adults, aged between 3 and 13 years. Classic VRA, with clinical significance, are usually identified much earlier, as persistent regurgitation often becomes evident after switching to solid food [1]. In contrast, an anatomical abnormality that causes only mild compression may go undetected for years and is incidentally discovered following advanced imaging investigations performed for another condition. Current results, corroborated previously reported adult-onset cases [6,17], indicate that the identification of an ARSA-type abnormality in an adult dog should not be considered unexpected and that age, in itself, cannot determine whether the anomaly is clinically relevant.

Several limitations of the present study should be acknowledged. First, the small number of canine patients precluded meaningful statistical analysis and limited the possibility of drawing robust conclusions regarding the clinical relevance of the observed findings. In addition, the exploratory nature of the case series limits the generalizability of the results and warrants cautious interpretation. Second, the gastrointestinal history was based on information provided by the owners; in one case, reliable information regarding the dog’s gastrointestinal history was unavailable. Third, the frequency, timing and characteristics of regurgitation were not prospectively documented using a standardised protocol. Fourth, the dogs underwent CT examination for clinical indications unrelated to this condition and no oesophageal-specific functional investigation (such as videofluoroscopic examination of swallowing or standardised contrast esophagography) was performed to determine whether the slight anatomical compression affected the transit of the food bolus. Fifth, since a control group of comparable dogs without ARSA was not evaluated, the incidence of intermittent regurgitation present in the history of patients in this series cannot be specifically attributed to the vascular abnormality. Finally, the results of the CT examination objectively allow the morphology of the oesophagus to be analysed at a given time, but cannot exclude the existence of intermittent functional disorders.

Despite these limitations, the present series provides additional information on a relatively poorly characterised canine vascular anomaly. Its specific contribution is not to demonstrate that ARSA causes regurgitation, but rather to identify a consistent pattern in adult dogs in which an isolated ARSA anomaly was associated with mild focal oesophageal compression without secondary oesophageal dilation, while most dogs had a history of intermittent regurgitation reported by their owners. This combination of findings lies in an intermediate zone, between clearly incidental ARSA without oesophageal involvement and clinically manifested vascular ring disease accompanied by significant oesophageal disorder.

## 5. Conclusions

In conclusion, isolated ARSA represented an incidental identification on CT examination in all eight adult canine patients and was consistently associated with mild focal oesophageal compression without being accompanied by oesophageal dilation or other vascular abnormalities of the aortic arch. Intermittent or rare regurgitation was reported by the owners in six dogs (75%); however, a causal relationship with the vascular abnormality could not be established. These results suggest that mild oesophageal compression associated with ARSA may require clinical attention, even in the absence of clear evidence of oesophageal obstruction on CT examination.

## Figures and Tables

**Table 1:** Clinical characteristics and indications for CT examination.

Case	Breed (Case Number)	Age (years)	Sex/Reproductive Status	Body Weight (kg)	Clinical Presentation and Reason for CT Examination	Main CT Findings Related to the Primary Clinical Problem
1	Border Collie (Case 1)	11	M/N	19	Mild dorsal nasal deformity, dyspnea and epistaxis; CT of the skull, cervicothoracic spine and thorax	Rostromedial nasal/sphenoidal mass with adjacent bone lysis; partial cribriform plate involvement; cranial mediastinal lymph node reactivity
2	GSD cross (Case 2)	3	F/S	30	Kyphosis, paraparesis and proprioceptive ataxia; CT scan of the thoracolumbar spine	Partial intervertebral disc mineralization at T7-T13 and disc extrusion at T12-T13
3	Bichon (Case 3)	6	F/I	6	Kyphosis, paraparesis and intermittent proprioceptive ataxia; CT scan of the cervicothoracolumbar spine	Degenerative spondylosis and L4-L5 disc protrusion
4	French Bulldog (Case 4)	8	F/S	11	Thoracolumbosacral pain; posture: kyphosis and wide hind-limb stance; gait: kyphosis, paraparesis with a tendency to drag the hind-limb toes, hypometria (scuffed nails) of the right hind limb; delayed proprioception on paw repositioning (knuckling test), flexion–extension (extensor postural thrust), and hopping in the hind limbs, and the right hind limb is more severely affected; panniculus reflex: reduced on the left side, absent on the right side; perianal reflex: reduced; CT scan of the cervicothoracolumbar spine	Multiple congenital vertebral malformations, thoracolumbosacral spondylosis deformans, L2-L4 intervertebral disc protrusion with mild spinal cord compression and severe stenosis of the right intervertebral foramen; mineralization of the intervertebral discs at T1-T2, T6-T8, T10-T11, and L7-S1
5	Boxer (Case 5)	9	F/S	29	Exercise intolerance (over the last 2–3 weeks), which has worsened over time; short, intense, dry cough; pleurodynia; and pleural friction rub on thoracic auscultation; CT scan of the chest	Small pleural effusion, subpleural bleb in the right caudal lung lobe, pulmonary atelectasis, and pulmonary fibrosis
6	Border Collie (Case 6)	13	M/I	17	Decreased appetite, respiratory signs (fatigue upon exertion, short bouts of coughing, harsh vesicular breath sounds on auscultation), lameness in the right forelimb and stiffness gait on both limbs; CT scan of the thoracoabdominal region	Pulmonary consolidation in the ventral area of the left cranial lung lobe, at the level of ribs 3–5; degenerative joint changes (right shoulder joint and hip joints, bilaterally)
7	American Staffordshire Terrier (Case 7)	5	M/I	31	Exercise intolerance, reluctance to move, and adoption of sterno-abdominal recumbent position after a few steps; pain and vocalisation upon palpation of the thorax or when attempting to lift the patient from the recumbent position; CT scan of the cervicothoracic region	L2-L3 intervertebral disc protrusion; small pleural effusion, uneven pleural thickening, and mild right lung atelectasis
8	French Bulldog (Case 8)	9	F/I	12	Progressive respiratory signs, more pronounced during the last month of life, nocturnal snoring, and intermittent nasal discharge of varying consistency (seromucous, very rarely reddish in colour); CT scan of the skull and thorax	Nasopharyngeal mass (1.5 × 2.6 × 0.9 cm) in the caudal nasopharynx, adherent to the dorsal wall, causing stenosis and exerting a mass effect on the soft palate (which is displaced ventrally) without affecting adjacent bony structures; elongation and thickening of the soft palate consistent with BOAS

**Table 2:** CT characteristics of ARSA and owner-reported gastrointestinal history.

In the Case	ARSA Characteristics *	Owner-Reported Gastrointestinal History
1. Case 1	As described below *	Rare, intermittent postprandial regurgitation reported since approximately 6–7 months of age, which became increasingly infrequent over time; the clinical examination performed at the time revealed no abnormalities in the upper segments of the digestive tract.
2. Case 2		The animal was adopted two years prior to this study, and the owner could not provide information regarding the presence of functional digestive disorders.
3. Case 3		The occurrence of rare episodes of regurgitation was noted, previously attributed to rapid eating (tachyphagia); no other functional abnormalities of the digestive system were identified during previous clinical examinations.
4. Case 4		Infrequent episodes of regurgitation since approximately 8 months of age), previously attributed to gastroesophageal reflux, as the patient ate only once a day and exhibited rapid eating; the patient was treated for this condition at the time and the clinical signs resolved; subsequently, further episodes of regurgitation were observed but occurred at long intervals and were not investigated further.
5. Case 5		The patient’s history revealed no signs characteristic of a significant digestive disorder.
6. Case 6		Episodes of digestive involvement (vomiting, diarrhoea) were reported during the patient’s early years, as well as transient episodes of regurgitation; these were treated symptomatically at the time.
7. Case 7		Previous episodes of regurgitation, vomiting and diarrhoea, particularly after access to inappropriate food (especially after walks in the park, when the patient would eat whatever it found on the street).
8. Case 8		The owner does not recall significant clinical signs involving the digestive tract, with the exception of rare episodes of regurgitation attributed to the patient’s brachycephalic conformation, which were treated symptomatically at the time.

* Common CT characteristics: in all eight dogs, the ARSA originated directly from the aortic arch and followed a retroesophageal course around the trachea and oesophagus before continuing towards the right thoracic limb. Mild oesophageal compression was present at the crossing site in all dogs. No oesophageal dilation cranial or caudal to the crossing site and no intraluminal gas or fluid were identified. No additional aortic arch vascular anomalies were detected.

## Data Availability

The data supporting the findings of this study were obtained from the medical records of canine patients examined at Link-Vet Bucharest between 2020 and 2025. Due to the inclusion of individual clinical and imaging information from animal medical records, the underlying patient-level data are not publicly available. De-identified data may be made available by the corresponding author upon reasonable request, subject to applicable privacy and confidentiality requirements.

## Abbreviations

The following abbreviations are used in this manuscript:

ARSA	Aberrant right subclavian artery
BCT	Brachiocephalic trunk
BOAS	Brachycephalic obstructive airway syndrome
CT	Computed tomography
F	Female
GSD	German Shepherd Dog
I	Intact
LSA	Left subclavian artery
M	Male
N	Neutered
RSA	Right subclavian artery
S	Spayed
T	Trachea
VRA	Vascular ring abnormalities
